# P-709. Risk factors for hypoxia and mechanical ventilation in Respiratory Syncytial Virus (RSV) infections in hospitalized solid organ transplant recipients (SOTR)

**DOI:** 10.1093/ofid/ofae631.905

**Published:** 2025-01-29

**Authors:** Maria A Mendoza, Victoria G Hall, Mitchell Dumais, Raul Rodríguez, Beatrice Z Sim, Alabdely Mayyadah, Michelle Yong, Zachary Yetmar, Raymund R Razonable, Yoichiro Natori

**Affiliations:** University of Utah, Salt Lake City, Utah; Peter MacCallum Cancer Centre, Toronto, Ontario, Canada; Mayo Clinic Rochester, Rochester, Minnesota; Miami Transplant Institute, Miami, Florida; Peter MacCallum Cancer Centre, National Centre for Infections in Cancer, University of Melbourne, Melbourne, Victoria, Australia; Cleveland Clinic, Cleveland, Ohio; Peter MacCallum Cancer Centre, Toronto, Ontario, Canada; Cleveland Clinic, Cleveland, Ohio; Mayo Clinic, Rochester, Minnesota; University of Miami, Miami Transplant Institute, Jackson Health System, Miami, Florida

## Abstract

**Background:**

RSV is a common respiratory infection that causes significant morbidity in SOTR. However, risk factors for complications such as hypoxic respiratory failure, mechanical ventilation, and secondary infections have not been well described.

**Figure:**
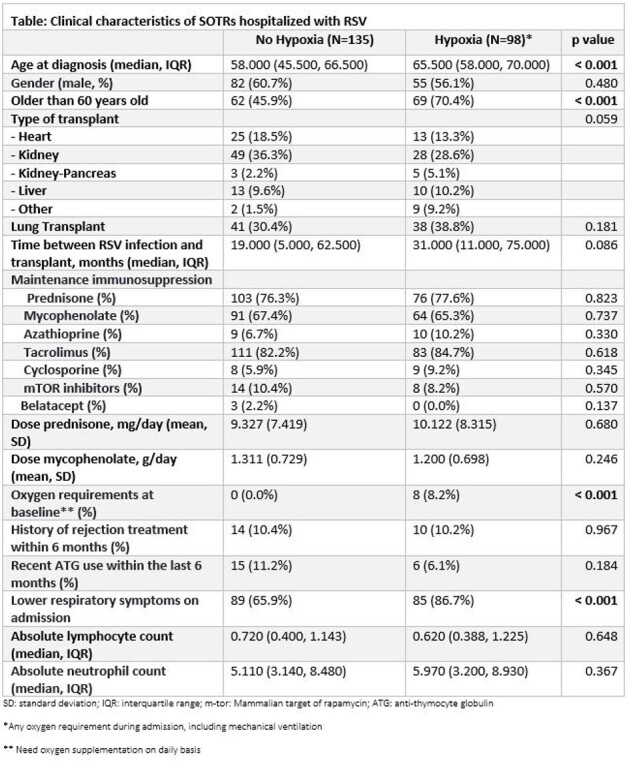

**Methods:**

This retrospective, international, multicenter cohort study is actively recruiting SOTR hospitalized with RSV. Here, we analyzed data from Mayo Clinic (Rochester, Florida, Arizona), Miami Transplant Institute, Cleveland Clinic, and Royal Melbourne Hospital between 2016 and 2019. Secondary infection was defined as culture positive infection within 90 days of RSV diagnosis. Whenever appropriate, logistic and Cox regression analysis were conducted to identify risk factors. Survival functions were estimated using the Kaplan-Meier method.

**Figure 1:**
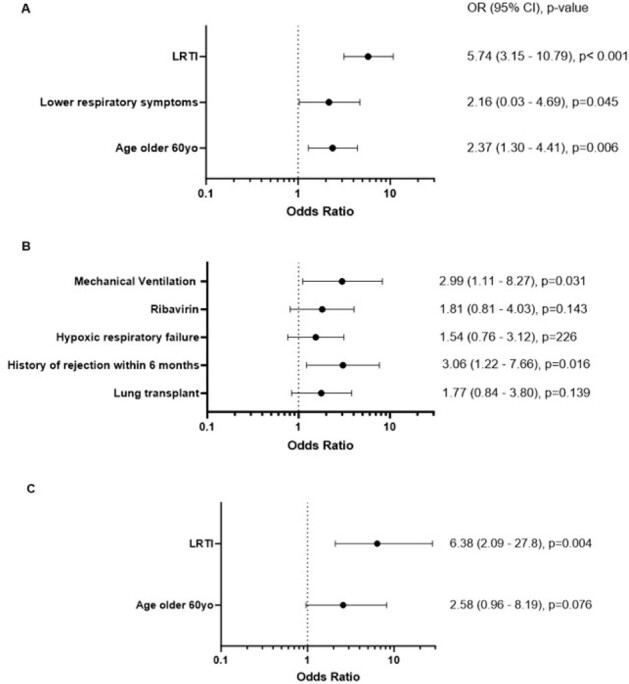
Forest plots of multivariable logistic regression models for risk factors for A) hypoxia, B) secondary infections, C) mechanical ventilation. Note: all variables included are the ones statistically significant on univariate analysis. Abbreviations: CI, confidence interval; OR, Odds ratio; LRTI: lower respiratory tract infection.

**Results:**

233 SOTRs (58.8% male; median, 61 years), including 77 (49.1%) kidney, 23 (9.9%) liver, 79 (33.9%) lung, and 38 (16.3%) heart transplants (Table). Hypoxic respiratory failure, mechanical ventilation, and secondary infection were seen in 98 (42.1%), 23 (9.9%), and 58 (24.9%) patients, respectively.

After multivariate analysis, age >60 years, symptoms and imaging evidence of lower respiratory tract infection (LRTI) were associated with oxygen requirement (Figure 1A); similar results were observed in an analysis of the lung transplant cohort only.

Risk factors on multivariate analysis for secondary infection include a history of rejection within six months and mechanical ventilation (Figure 1B).

Risk factors for mechanical ventilation included age >60 years and LRTI on imaging (Figure 1C) after multivariate analysis.

Seventeen (7.3%) patients died within 90 days of diagnosis. Cox univariate analysis identified age >60 years and LRTI on imaging, secondary infections, mechanical ventilation and hypoxia on admission were associated with higher mortality (Figure 2 and 3).

**Figure 2:**
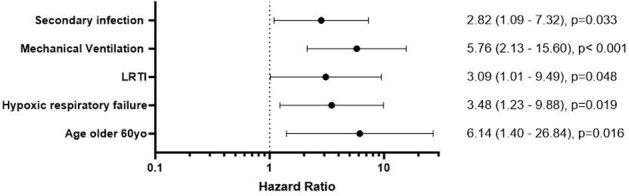
Forest plot with Cox proportional hazards models. Note: only univariate results shown, multivariate not done due to low number of events.

**Conclusion:**

This multicenter study of SOTR hospitalized with RSV identified older age ( >60 years old) as a risk factor for hypoxic respiratory failure, mechanical ventilation and death. Mechanical ventilation and a history if rejection within six months of RSV were associated with increased risk of secondary infections, identifying them as high-risk that needs close surveillance for this complication.

**Figure 3:**
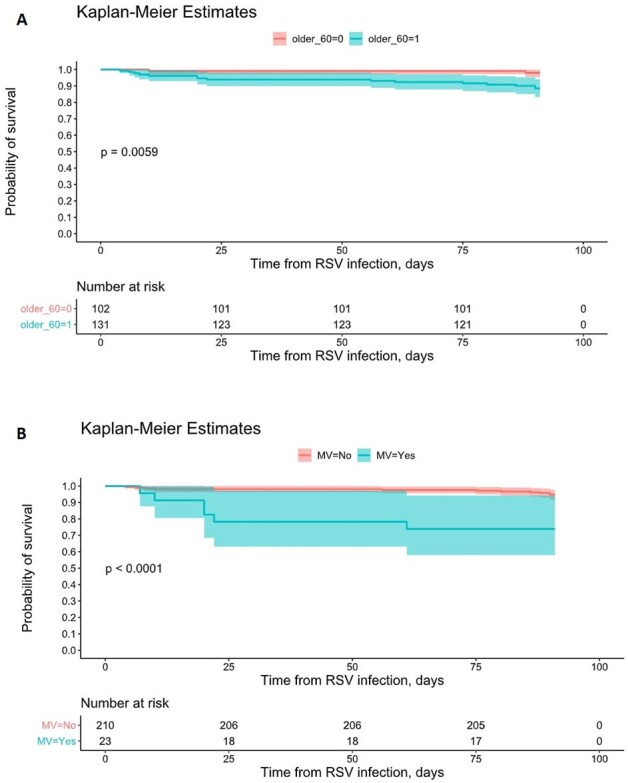
Effects of A) Age older than 60 years old and B) mechanical ventilation on 90 day probability of survival after RSV infection

**Disclosures:**

**Michelle Yong, MBBS, FRACP, MPH, PhD**, MSD: Grant/Research Support|MSD: Honoraria|Takeda: Advisor/Consultant **Yoichiro Natori, MD**, Eurofin/Viracor: Honoraria|Nobel Pharma: Advisor/Consultant

